# Checkpoint-enriched T cells with retained effector-associated features are associated with disease activity in systemic lupus erythematosus

**DOI:** 10.3389/fimmu.2026.1818795

**Published:** 2026-09-01

**Authors:** Paola Santana-Sánchez, Julián A. Gajón, Astrid Asminda Ramírez-Pérez, María Victoria Legorreta-Haquet, Luis Manuel Amezcua-Guerra, Laura C. Bonifaz, Adriana Karina Chávez-Rueda

**Affiliations:** 1 Doctorado en Ciencias Biológicas y de la Salud, Universidad Autónoma Metropolitana, Mexico City, Mexico; 2 Unidad de Investigación Médica en Inmunología, Unidad Médica de Alta Especialidad (UMAE) Hospital de Pediatría, Centro Médico Nacional Siglo XXI, Instituto Mexicano del Seguro Social, Mexico City, Mexico; 3 Posgrado en Ciencias Bioquímicas, Universidad Nacional Autónoma de México, Mexico City, Mexico; 4 Servicio de Reumatología, Unidad Médica de Alta Especialidad (UMAE) Hospital de Especialidades, Centro Médico Nacional Siglo XXI, Instituto Mexicano del Seguro Social, Mexico City, Mexico; 5 Departamento de Inmunología, Instituto Nacional de Cardiología Ignacio Chávez, Mexico City, Mexico; 6 Unidad de Investigación Médica en Inmunoquímica, Unidad Médica de Alta Especialidad (UMAE) Hospital de Especialidades, Centro Médico Nacional Siglo XXI, Instituto Mexicano del Seguro Social, Mexico City, Mexico; 7 Coordinación de Investigación en Salud, Centro Médico Nacional Siglo XXI, Instituto Mexicano del Seguro Social, Mexico City, Mexico

**Keywords:** autoimmunity, chronic immune activation, immune checkpoint receptors, systemic lupus erythematosus, T cells

## Abstract

Systemic lupus erythematosus (SLE) is a chronic autoimmune disease characterized by sustained immune activation and fluctuating disease activity. Although exhaustion-associated markers are frequently detected on T cells from patients with SLE, the biological significance of these signatures remains poorly defined. Here, we performed high-dimensional phenotypic profiling of circulating CD8^+^ and CD4^+^ T cells from patients with inactive and active SLE using spectral flow cytometry, complemented by an *in vitro* model of sustained TCR stimulation. We identified distinct T cell subsets characterized by coordinated expression of multiple immune checkpoint receptors, including PD-1, LAG-3, TIM-3, TIGIT and CD39, together with the transcriptional regulator TOX. Notably, checkpoint-enriched populations were preferentially expanded in patients with active disease (aSLE). Despite the accumulation of inhibitory features, both CD8^+^ and CD4^+^ T cells retained effector-associated features, including cytokine production and cytotoxic mediator expression, suggesting that immune checkpoint expression in this context is not uniformly associated with the complete loss of effector-associated features characteristic of canonical T cell exhaustion. Trajectory inference of *ex vivo* datasets, together with the *in vitro* model, suggested an association between sustained activation and increasing immune checkpoint receptor expression while maintaining the expression of effector-associated molecules. Collectively, these findings identify distinct checkpoint-enriched T cell phenotypic states in SLE, in which immune checkpoint receptor expression coexists with retained effector-associated features. Overall, this work provides a framework for interpreting immune checkpoint-associated T cell phenotypes in systemic autoimmunity.

## 1 Introduction

Systemic lupus erythematosus (SLE) is a complex autoimmune disease characterized by chronic systemic inflammation that affects multiple organs (1). Its pathogenesis involves broad dysregulation of the immune system, including abnormal activation of B and T cells, leading to excessive production of autoantibodies directed against nuclear components (2, 3). Despite advances in understanding disease mechanisms, therapeutic responses remain heterogeneous, and a significant proportion of patients fail to achieve long-term remission.

Current therapies can temporarily reduce disease activity, but most patients with SLE eventually relapse and require retreatment or alternative immunosuppressive agents. Consequently, increasing attention has been given to novel cell-based therapies aimed at restoring immune balance in autoimmune diseases. Since 2019, CD19-targeted CAR-T-cell therapies have been evaluated to deplete autoreactive B cells and ameliorate clinical manifestations (4–9). Although these approaches primarily target B cells, their efficacy depends on the functional properties of genetically modified T cells, renewing interest in T cells as therapeutic targets in SLE immune dysregulation. In this context, CAR-T-cell therapies exploit the cytotoxic and immunomodulatory capacities of T cells to reprogram pathogenic immune networks and restore self-tolerance (10, 11).

In SLE, loss of immune tolerance promotes aberrant CD4^+^ T cell responses that provide sustained help to autoreactive B cells through costimulatory interactions and cytokine secretion. In parallel, continuous antigen exposure and a proinflammatory milieu drive persistent activation of CD8^+^ T cells, which infiltrate target tissues and contribute to local inflammation and tissue damage (12, 13). As a result, subsets of both CD4^+^ and CD8^+^ T cells are embedded in self-amplifying inflammatory networks characterized by chronic immune activation and sustained TCR signaling, conditions that promote progressive functional adaptation toward T cell exhaustion. Exhausted T cells (Tex) are defined by sustained expression of immune checkpoint inhibitory receptors, such as PD-1, TIM-3 and LAG-3, together with impaired effector function and proliferative capacity. This state is associated with the upregulation of the transcription factor TOX (14–16).

Although T cell exhaustion was initially described in chronic infections and cancer, exhaustion-associated phenotypes have also been reported in SLE. CD8^+^ T cells exhibiting exhaustion-related transcriptional profiles have been identified (17). Interestingly, exhaustion-associated programs in SLE appear to be context dependent: while exhaustion is classically linked to functional impairment, immune checkpoint-expressing CD4^+^ and CD8^+^ T cell populations have been observed both in patients in remission (18) and in settings of active disease. Inhibitory receptor-expressing T cells have also been detected within inflamed tissues and autoreactive subsets in active lupus, including CCR7^low^ CD74^high^ CD4^+^ T cells displaying IFN I expression and immunologically active features (19), as well as renal-infiltrating T cells in lupus-prone MRL/lpr mice (20). However, most studies rely on limited marker panels rather than comprehensive multiparameter approaches, limiting the ability to distinguish bona fide exhaustion from sustained activation or adaptive inhibitory programs.

Accordingly, the precise nature of T cell alterations in SLE remains unresolved. Defining whether inhibitory receptor-expressing T cells represent activation states, checkpoint-modulated adaptations, or bona fide exhaustion is particularly important, as populations coexpressing inhibitory receptors and effector molecules may reflect highly activated or adaptively constrained immune states rather than terminally exhausted cells (21). Taken together, these observations underscore the need for a comprehensive characterization of T cell heterogeneity in SLE, as distinct immune states may differentially influence disease activity and therapeutic response. Therefore, in this study, we employed high-dimensional spectral flow cytometry combined with unsupervised approaches to characterize circulating CD8^+^ and CD4^+^ T cell subpopulations in patients with active (aSLE) and inactive (iSLE) disease compared with those in healthy donors (HD). We identified clusters defined by coordinated expression of immune checkpoint-associated programs, including PD-1, LAG-3 and TIM-3, together with transcriptional regulators such as TOX and TCF1, that nonetheless coexpressed cytotoxic mediators (Perforin and Granzyme B) and proinflammatory cytokines (IFNγ and TNFα). Collectively, these findings identify checkpoint-modulated T cell phenotypic states associated with disease activity and characterize their phenotypic organization through trajectory inference, providing insight into T cell heterogeneity in systemic autoimmunity.

## 2 Materials and methods

### 2.1 Participant recruitment

The patient cohort (n = 55) has been previously described (22), including its demographic, clinical and treatment characteristics; treatment information is also summarized in **Supplementary Table 4**. The analyses presented here address distinct immunophenotypic questions. Briefly, patients were recruited from the rheumatology service at Hospital de Especialidades, Centro Medico Nacional Siglo XXI, IMSS (Mexico City, Mexico) and fulfilled the 2019 EULAR/ACR Classification Criteria for systemic lupus erythematosus (SLE).

Disease activity was determined using the SLEDAI-2K score, with patients categorized as active (aSLE, SLEDAI-2K ≥ 4; n=19) or inactive (iSLE, SLEDAI-2K < 4; n=36) (23, 24). Demographic variables (sex, age, and age at diagnosis) and complete clinical records were obtained, with median ages (interquartile range) of 34 (28–51) years for aSLE and 36 (30–44) years for iSLE. Exclusion criteria included concomitant autoimmune diseases, infections, malignancies, or recent changes in immunosuppressive therapy.

For the present study, the same patient cohort was analyzed, and an additional control group consisting of 12 newly recruited healthy donors (HD) was included. Demographic characteristics were comparable to those previously reported, with a median (interquartile range) age of 26 (25–53) years and a female-to-male ratio of 9:1. All participants provided written informed consent, and the study was approved by the IMSS Ethics Committee (R-2022-785-047).

### 2.2 PBMC collection

Peripheral blood samples (15 mL) were obtained from each participant in EDTA tubes. Mononuclear cells were isolated by density-gradient centrifugation using Lymphoprep (Serumwerk, Bernburg, Germany) at 400× g for 30 min. The interphase containing Peripheral Blood Mononuclear Cells (PBMCs) was collected, washed, and stored in cryopreservation medium until use.

### 2.3 *Ex vivo* CD4^+^ and CD8^+^ T cell staining

PBMCs were thawed and stained with a fixable viability dye, followed by surface staining with antibodies for 20 min at 4 °C in the dark. After washing with FACS buffer, cells were permeabilized using the Transcription Factor Staining Buffer Set (BioLegend, San Diego, CA, USA) for 45 min at room temperature and subsequently stained intracellularly for 30 min. The complete list of reagents, including clones and conjugates, is provided in **Supplementary Table 1**. Samples were acquired on a Cytek Aurora 3L spectral flow cytometer (Cytek Biosciences, Fremont, CA, USA) and data were analyzed using FlowJo v10.9.0 (BD Biosciences, Ashland, OR USA). The gating strategy included exclusion of doublets, removal of non-viable cells and identification of CD4^+^ and CD8^+^ T cells (**Supplementary Figure 1A**). Representative fluorescence minus one (FMO) controls were included to support gating of markers with continuous or low-level expression (**Supplementary Figure 1B**).

### 2.4 *In vitro* T cell stimulation

From the same thawed PBMC samples, another fraction of the cells was cultured in complete RPMI 1640 medium supplemented with 10% fetal bovine serum (FBS) and 1% antibiotic-antimycotic (Gibco, Thermo Fisher Scientific). T cells were stimulated for a total of 7 days with plate-bound anti-CD3 (1.3 μg/mL, BioLegend) and soluble anti-CD28 (2.5 μg/mL, BioLegend) every third day. Cells were restimulated twice under the same conditions. For the final stimulation, only anti-CD3 was used to mimic chronic T cell receptor (TCR) engagement in the absence of co-stimulation. At the end of the culture period, cells were restimulated with cell stimulation cocktail (Cytek Biosciences, Fremont, CA, USA) and protein transport inhibitor cocktail (Invitrogen, Thermo Fisher Scientific) for 6 h to allow intracellular cytokine accumulation. Following *in vitro* stimulation, cells were stained using the same antibody panel employed in the *ex vivo* analysis. Cultures were maintained at 37 °C in 5% CO_2_ until collection for flow cytometry analysis.

### 2.5 Degranulation assay

PBMCs were thawed and cultured in complete RPMI 1640 medium supplemented with 10% fetal bovine serum (FBS) and 1% antibiotic-antimycotic (Gibco, Thermo Fisher Scientific). Cells were stimulated for 1 h with cell stimulation cocktail (Cytek Biosciences, Fremont, CA, USA), followed by the addition of protein transport inhibitor cocktail (Invitrogen, Thermo Fisher Scientific) for an additional 6 h. At the end of the incubation period, cells were stained with a fixable viability dye and surface antibodies against CD3, CD4, CD8, CD45RO, CD69, TIM-3, LAG-3, PD-1 and TIGIT. Degranulation was assessed by surface staining for CD107a (LAMP-1) and CD107b (LAMP-2). Intracellular staining was performed for TCF1 and TOX. Intracellular cytokines and cytotoxic mediators, including IFNγ, TNFα, Granzyme B and Perforin were also included. The complete list of reagents, including clones and fluorochrome conjugates, is provided in **Supplementary Table 1**. Live, singlets, CD4^+^ and CD8^+^ T cells were analyzed using FlowJo v10.9.0 (BD Biosciences, Ashland, OR USA).

### 2.6 Unsupervised flow cytometry analysis

Single-cell events (singlets) were first identified to exclude doublets, followed by gating on live cells and subsequent identification of CD4^+^ and CD8^+^ T cells using FlowJo v10.9.0 (BD Biosciences, Ashland, OR USA). Events were concatenated across samples within each group using all fluorescence parameters after spectral unmixing and subjected to dimensionality reduction using the Uniform Manifold Approximation and Projection (UMAP) plugin (v4.1.1) with 15 nearest neighbors, a minimum distance of 0.5 and the Euclidean distance metric. The resulting embeddings were visualized as two-dimensional projections. Clustering was subsequently performed using the FlowSOM plugin (v4.1.0) with an eight-cluster configuration and a 10 × 10 grid. Several clustering resolutions were explored during analysis. The final eight-cluster configuration was selected because it preserved biologically distinct populations while avoiding over-fragmentation of phenotypically similar subsets. Final cluster identities were defined through supervised analysis based on marker expression patterns observed in the UMAP space, using FlowSOM outputs as reference. UMAP coordinates and final cluster assignments were exported to R (v4.4.3, RStudio) for downstream statistical analysis and visualization.

To minimize biases due to unequal cell numbers across samples, similar numbers of events were acquired for each sample and equal downsampling was applied prior to unsupervised analysis. The resulting concatenated datasets were used for UMAP visualization, clustering and identification of candidate phenotypic populations. Following clustering, the corresponding immunophenotypic populations were translated into supervised gating strategies and quantified independently in each donor. The frequencies of these supervised populations were then calculated independently for each donor and all statistical analyses were subsequently performed using donor-level measurements, with individual donors considered the independent biological replicates.

### 2.7 Downstream analysis and visualization

Relative cluster distributions derived from the concatenated datasets were used for visualization of group-level differences for each study group (HD, iSLE and aSLE). Relative cluster distributions were visualized using stacked barplots with cluster identities represented as proportions within each disease group using ggplot2 (v3.5.2). Data manipulation was performed using dplyr (v1.1.4) and tidyr (v1.3.1) and percentage scaling was applied with scales (v1.3.0). Dimensionality reduction results were visualized as two-dimensional UMAP projections, in which individual cells were colored according to their assigned cluster identity. Marker expression values were normalized by Z-score transformation and mean fluorescence intensity (MFI) values were calculated for each marker within each cluster. Heatmaps of Z-score-normalized MFI values were generated using pheatmap (v1.0.13), applying a fixed cluster order. To illustrate functional relationships between clusters and immunophenotypic markers, chord diagrams were generated using the circlize package (v0.4.16).

### 2.8 Pseudotime analysis and marker dynamics

Pseudotime inference was performed with the Slingshot package (v2.14.0, Bioconductor), with UMAP coordinates used as input and manually defined clusters as labels within the Single Cell Experiment framework. Cluster 3 was specified as the analytical reference (root) population for trajectory inference based on its relatively low expression of effector molecules and immune checkpoint receptors compared with the remaining clusters. Pseudotime values for each trajectory were extracted and visualized on the UMAP embedding. To evaluate marker dynamics along pseudotime, expression values of immunophenotypic markers were standardized by Z-score transformation within each trajectory. Cells were ordered according to pseudotime, and the resulting Z-score values were visualized as heatmaps using pheatmap (v1.0.13). Functional annotation of markers (memory/stemness, effector and checkpoint-enriched) was incorporated into the heatmaps to facilitate biological interpretation. Z-score normalization was used exclusively for visualization purposes and to facilitate comparison among markers with different dynamic ranges. Therefore, Z-score values represent relative expression patterns of each marker across the analyzed dataset and should not be interpreted as absolute fluorescence intensities.

### 2.9 Linear discriminant analysis

Linear discriminant analysis (LDA) was performed using selected*ex vivo* CD8^+^ T cell immunological variables derived from the supervised analyses (**Supplementary Table 3**). Variables were standardized to unit variance prior to analysis. LDA was implemented in R (v4.4.3, RStudio) using the MASS package. The resulting discriminant functions represent linear combinations of the selected variables that maximize separation among the study groups.

To examine the association between the integrated immunological signature and continuous disease activity, LD1 scores derived from the LDA model were correlated with SLEDAI-2K scores using Spearman’s rank correlation. This analysis included only patients with SLE. Healthy donors were excluded.

### 2.10 Statistical analysis

Normality of data distribution was assessed using the Shapiro-Wilk test. Comparisons among groups were performed using the Kruskal-Wallis test, followed by Dunn’s multiple comparison *post hoc* test. Correlations between variables were analyzed using the nonparametric Spearman test. All statistical analyses were performed using GraphPad Prism software v8.0 (GraphPad Software, Inc., La Jolla, CA, USA). A p-value of <0.05 was considered statistically significant. For the exploratory analyses performed on concatenated datasets, differences in cluster distributions among groups (HD, iSLE and aSLE) were assessed in R using Fisher’s exact test for pairwise comparisons (HD vs iSLE, HD vs aSLE and iSLE vs aSLE). P-values were adjusted for multiple testing using the Benjamini-Hochberg false discovery rate (FDR) method. Clusters with adjusted p < 0.05 were considered significant, regardless of the absolute magnitude of change.

## 3 Results

### 3.1 CD8^+^ T cells in SLE display heterogeneous immune checkpoint receptor and effector programs, as revealed by high-dimensional analysis

**Figure 1:**
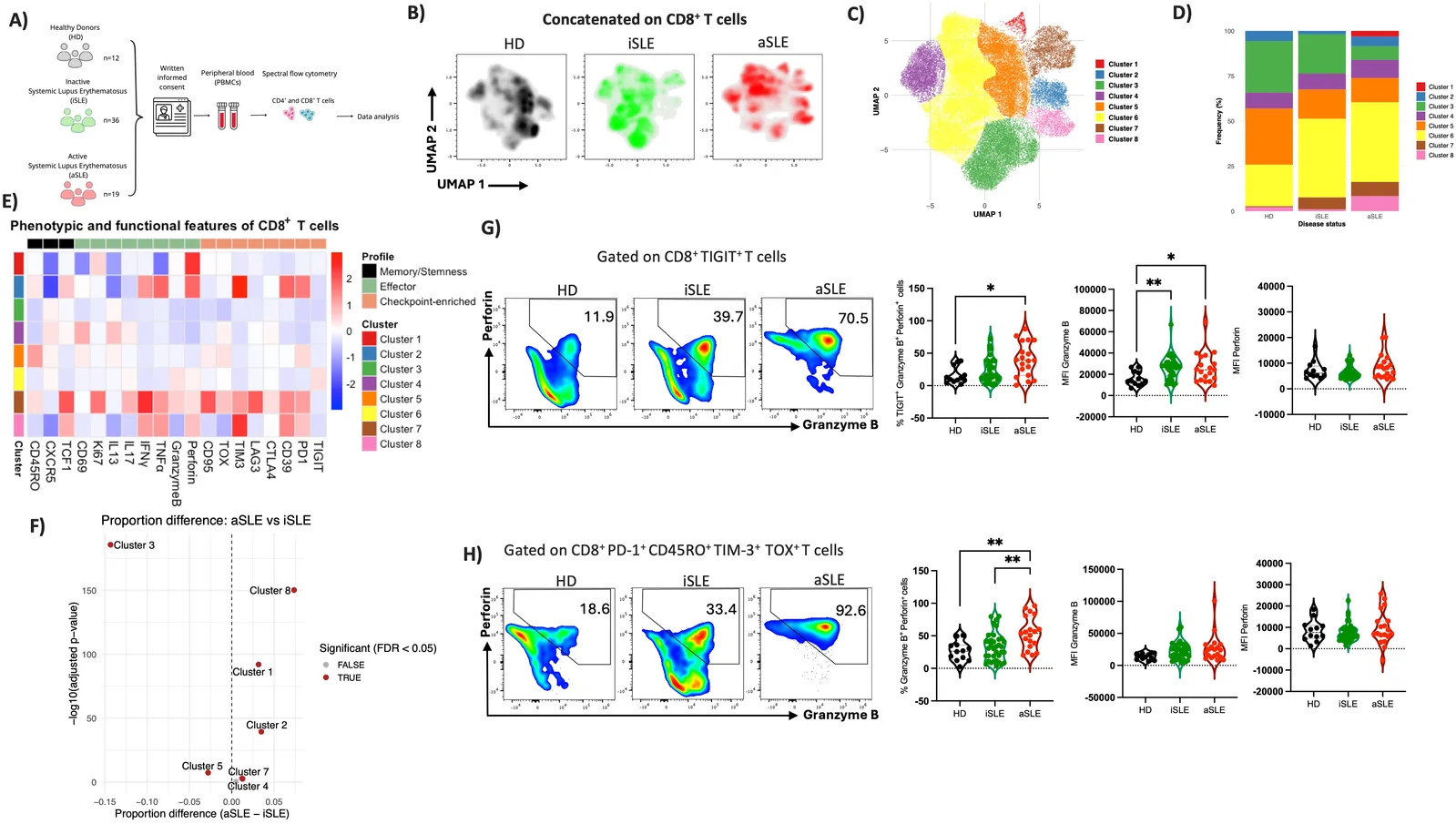
High-dimensional analysis revealed heterogeneous immune checkpoint receptor expression and retained cytotoxic programs in CD8^+^ T cells from patients with SLE. **(A)** Peripheral blood mononuclear cells (PBMCs) from healthy donors (HD; n=12), patients with inactive SLE (iSLE; n=36), and patients with active SLE (aSLE; n=19) were analyzed by high-dimensional spectral flow cytometry. **(B)** UMAP projections of concatenated CD8^+^ T cells illustrate the global phenotypic landscape across disease states. **(C)** Clustering revealed eight distinct CD8^+^ T cell clusters visualized on the UMAP embedding and color coded by cluster identity. **(D)** Relative cluster frequencies across disease activity states. **(E)** Heatmap of z score-normalized marker expression highlights the phenotypic and functional heterogeneity across clusters, including coordinated expression of immune checkpoint receptors and effector-associated molecules. **(F)** Differential cluster abundance between patients with aSLE and patients with iSLE is shown as proportion differences with false discovery rate (FDR)-adjusted p values. **(G)** Representative dot plots show Granzyme B and Perforin expression within TIGIT^+^ CD8^+^ T cells with corresponding quantification of frequencies and median fluorescence intensity (MFI) shown in adjacent panels. **(H)** Representative dot plots show PD-1^+^CD45RO^+^ TIM-3^+^ TOX^+^ CD8^+^ T cells with corresponding quantification of frequencies and MFI shown in adjacent panels. Data are presented as individual values with median lines. Statistical significance was assessed using the Kruskal-Wallis test followed by Dunn’s *post hoc* multiple comparisons test (*p < 0.05; **p < 0.01; ***p < 0.001).

Previous studies have reported that the frequency of circulating CD8^+^ T cells correlates with disease activity in patients with SLE, and that expression of Granzyme B, granulysin and Perforin distinguishes active from inactive disease states (22). Building on these findings and other reports describing immune checkpoint receptor expression under conditions of chronic immune activation in patients with SLE (20, 25, 26), we performed a comprehensive phenotypic analysis of peripheral blood CD8^+^ and CD4^+^ T cells from patients with aSLE, iSLE and HD using high-dimensional spectral flow cytometry (**Figure 1A**). Given the complexity of the dataset, which comprised 23 phenotypic and functional markers, we applied unsupervised dimensionality reduction and clustering approaches to define the global CD8^+^ and CD4^+^ T cell landscape without imposing predefined gating strategies. This approach enabled the identification of distinct T cell programs that cannot be resolved using conventional two-dimensional flow cytometric analyses.

To explore phenotypic diversity within the CD8^+^ T cell compartment, we applied unsupervised analysis combining dimensionality reduction by uniform manifold approximation and projection (UMAP) followed by unsupervised clustering. Individual marker expression patterns projected onto the UMAP embedding are shown in **Supplementary Figure 2**. This analysis revealed different patterns between groups and eight distinct CD8^+^ T cell clusters (**Figures 1B, C**), consistent with previous reports that have described extensive CD8^+^ T cell heterogeneity under chronic inflammatory (27) and autoimmune disease conditions (28). A summary of the key markers defining each cluster and their corresponding biological annotations is provided in **Supplementary Table 2**. These clusters were characterized by coordinated immune checkpoint receptor expression and effector-associated features.

Comparative analysis revealed disease-associated CD8^+^ T cell clusters across disease groups (**Figures 1D, E**; **Supplementary Figure 3A**). Cluster 1, characterized by coexpression of Ki67 and Perforin, was detected exclusively in patients with aSLE, consistent with a proliferative cytotoxic subset that was enriched in patients with active disease. In contrast, Cluster 2, expressing CD45RO, IFNγ, TNFα, Perforin and multiple inhibitory receptors, including TIM-3, CD39, and PD-1, together with the transcriptional regulator TCF1, was reduced in patients with iSLE compared with that in both HD and patients with aSLE, suggesting differential representation of this checkpoint-enriched population during inactive disease. Cluster 3, defined by CXCR5^low^ expression, was reduced in patients with aSLE but preserved in patients with iSLE and HD, whereas Cluster 4, characterized by CXCR5, CD69, IL-13, and IL-17A^low^ expression, remained stable across all groups. In Cluster 5, the expression of CD45RO, CD69 ^low^ and IL-13 ^low^, together with that of CD95, TOX and PD-1, decreased in patients with SLE, indicating altered representation of these subsets. Collectively, these findings suggest that Clusters 3 and 5 are selectively perturbed in patients with SLE.

In contrast, Cluster 6, characterized by Granzyme B, Perforin, and TIGIT, was enriched in patients with active disease, indicating preferential representation of a predominantly cytotoxic CD8^+^ T cell population associated with active SLE.

Clusters 7 and 8 were distinguished by the coordinated expression of multiple immune checkpoint inhibitory receptors while coexpressing effector-associated molecules. Cluster 7 exhibited a complex phenotype characterized by expression of CD45RO and multiple immune checkpoint inhibitory receptors, including TIM-3, LAG-3, CTLA-4, CD39 and PD-1, together with CD95 and the transcriptional regulators TOX and TCF1, as well as effector mediators such as IL-17A, IFNγ, TNFα, Granzyme B and Perforin. Notably, this cluster also expressed Ki67, which is indicative of proliferative capacity, but lacked TIGIT, CXCR5, CD69 and IL-13. The coexistence of the progenitor-associated transcription factor TCF1 (29) with markers closely linked to terminal exhaustion (30, 31) suggests that Cluster 7 represents a checkpoint-enriched effector T cell phenotype characterized by the coexpression of immune checkpoint receptors and effector-associated molecules. Cluster 8 similarly displayed coexpression of IFNγ, TNFα and Perforin together with multiple checkpoint-enriched markers, including TIM-3, CD39 and PD-1, and the memory/stemness-associated transcription factor TCF1. This phenotype was consistent with retained effector-associated features, and Cluster 8 was expanded in patients with aSLE.

Exploratory comparison of cluster distributions revealed the selective expansion of cytotoxic subsets (Clusters 1 and 8) during active disease. In contrast, Clusters 3 and 5 were markedly reduced in patients with aSLE (**Figure 1F**; **Supplementary Figures 3B, C**). Given that Clusters 3 and 5 were preserved in HD and in patients with iSLE but selectively diminished during active disease, they represent CD8^+^ T cell states that are preferentially affected during aSLE. Notably, Cluster 2 was substantially reduced in patients with iSLE compared with both HD and patients with aSLE, indicating a disease state-specific modulation distinct from activity-dependent changes, whereas Cluster 4 remained stable across all three groups, suggesting relative resistance to disease-associated perturbations. Finally, multicheckpoint-enriched Cluster 7 was detected exclusively in SLE patients, whereas Cluster 8 further highlighted the accumulation of checkpoint-enriched CD8^+^ T cells retaining effector-associated marker expression in the context of systemic inflammation.

To further characterize the CD8^+^ T cell clusters identified by unsupervised analysis, we performed supervised quantification of selected markers associated with the phenotypes defined in each cluster (**Table 1**). Consistent with the phenotype of Cluster 1, we quantified the coexpression of Ki67 and Perforin in CD8^+^ T cells. Although no statistically significant differences were detected among groups, a trend toward increased frequencies and median fluorescence intensity (MFI) of both markers was observed in patients with aSLE compared with those with inactive disease (**Table 1**; **Supplementary Figure 3D**).

**Table 1:** Frequencies of CD8^+^ T cell subsets in SLE patients and HD.

Subset	Parameter	HD (Median; Q1-Q3)	iSLE (Median; Q1-Q3)	aSLE (Median; Q1-Q3)	p-Value
Cluster 1	% CD8^+^ Ki67^+^ Perforin^+^	12.34 (4.85-16.15)	9.22 (4.59-20.70)	15.80 (6.51-33.80)	0.19
	MFI Perforin	5710 (2643-8471)	4220 (2892-7629)	6721 (3999-12397)	0.18
	MFI Ki67	1784 (1705-1863)	1811 (1757-1871)	1858 (1753-2085)	0.36
Cluster 6	% CD8^+^ TIGIT^+^	12.65 (10.88-17.20)	23 (11.58-73.83)	18.20 (6.40-45.60)	0.07
	MFI TIGIT	19460 (16096-22442)	21843 (17009-27947)	15700 (12242-20042)	0.007**
	% Granzyme B^+^ Perforin^+^	10.77 (6.75-28.28)	17.05 (10.18-38.38)	39.10 (15.90-69.80)	0.01*
	MFI Granzyme B	13827 (10964-22402)	27818 (18556-30995)	24175 (14617-36542)	0.008**
	MFI Perforin	6095 (5052-8841)	5751 (4646-7573)	7837 (4671-10529)	0.43
	% CD8^+^ CD45RO^-^ PD-1^+^	6.60 (2.30-8.30)	7.32 (4.19-12.23)	10.70 (4.21-36.60)	0.11
	% CD8^+^ CD45RO^+^ PD-1^+^	14.60 (7.73-20.15)	10.70 (7.01-15.08)	10.40 (4.79-25.50)	0.70
Checkpoint-enrichment	% TIM-3^+^ TOX^+^	21.30 (3.18-54.60)	15.70 (2.75-32.73)	13.10 (3.14-41.60)	0.87
	% Granzyme B^+^ Perforin^+^	26.35 (13.25-35.70)	32.10 (13.80-44.13)	53.80 (33.80-80.90)	0.002**
	MFI Granzyme B	14058 (9655-18616)	22639 (14416-31943)	24050 (13228-32382)	0.054
	MFI Perforin	7626 (4829-10935)	7024 (4828-9289)	7160 (5727-12981)	0.62
	% CD8^+^ CD45RO^+^ PD-1^-^	21.25 (17.90-29.53)	16.65 (7.14-28.08)	16.20 (7.40-28.60)	0.23
	% CD8^+^ CD45RO^-^ PD-1^-^	56.65 (44.68-64.53)	61.85 (45.28-67.03)	55.20 (21.10-62.80)	0.34

Data are shown as median and interquartile range (Q1-Q3). The p-values reported in the table correspond to the overall Kruskal-Wallis test across the three groups (HD, iSLE and aSLE). Significant pairwise differences were assessed using Dunn´s multiple comparisons test. *p < 0.05; **p < 0.01. Subsets shown below CD8^+^ CD45RO^+^ PD-1^+^ were analyzed within this compartment. aSLE, active systemic lupus erythematosus; iSLE, inactive systemic lupus erythematosus; HD, healthy donors; MFI, median fluorescence intensity. Statistical significance is indicated by the asterisks.

We next examined markers associated with Cluster 6, focusing on the CD8^+^ TIGIT^+^ compartment. The frequency of CD8^+^ TIGIT^+^ T cells tended to increase in patients with SLE, with a greater representation in patients with iSLE than in patients with aSLE. However, a statistically significant difference in the MFI of TIGIT was detected between patients with active and inactive SLE, indicating higher TIGIT expression in the inactive disease group. In contrast, HDs did not display significant differences in either frequency or MFI of TIGIT expression (**Table 1**; **Supplementary Figure 3E**).

Assessment of cytotoxic mediator expression within the CD8^+^ TIGIT^+^ compartment revealed a significant increase in the proportion of Granzyme B^+^ Perforin^+^ cells in patients with aSLE compared with that in HDs, with a nonsignificant trend toward inactive disease. In parallel, the MFI of Granzyme B was significantly greater in both patients with active and inactive SLE than in HDs, whereas the MFI of Perforin remained unchanged across groups (**Table 1**; **Figure 1G**).

To further integrate and phenotypically validate CD8^+^ T cell populations characterized by the expression of multiple immune checkpoints, we performed a supervised analysis encompassing clusters enriched for inhibitory receptors. Based on the unsupervised clustering results, which consistently revealed the coexpression of CD45RO and PD-1 across multiple checkpoint-enriched clusters, we selected the CD8^+^ CD45RO^+^ PD-1^+^ compartment as a phenotypic anchor population for downstream analyses. Although a trend toward increased frequencies of CD8^+^ CD45RO^-^ PD-1^+^ T cells was observed in patients with aSLE compared with HDs, subsequent analyses were restricted to the CD45RO^+^ PD-1^+^ subset, as this population most closely recapitulated the phenotypic composition of the multicheckpoint clusters identified by unsupervised analysis (**Table 1**; **Supplementary Figure 3F**).

Within the CD8^+^ CD45RO^+^ PD-1^+^ compartment, we next evaluated the coexpression of TIM-3 and TOX, an inhibitory surface receptor and a transcriptional regulator, respectively (**Table 1**; **Supplementary Figure 3G**). Phenotypic assessment of the TIM-3^+^ TOX^+^ subset revealed a statistically significant increase in the frequency of Granzyme B^+^ Perforin^+^ cells in SLE patients compared with that in HDs, together with a trend toward higher Granzyme B and Perforin MFI (**Figure 1H**), indicating that retained effector-associated features are preferentially associated with antigen-experienced, multicheckpoint-expressing CD8^+^ T cells.

Taken together, these results demonstrate that CD8^+^ T cells in aSLE exhibit extensive immune checkpoint coexpression while retaining expression of cytotoxic and proliferation-associated markers.

### 3.2 Pseudotime inference suggested phenotypic trajectories toward multicheckpoint-enriched CD8^+^ T cell states in patients with SLE.

The coexistence of immune checkpoint receptor expression and effector-associated molecule expression across multiple CD8^+^ T cell clusters suggested phenotypic continuity across these populations. To explore this possibility, we next performed pseudotime inference to characterize potential phenotypic trajectories underlying the observed heterogeneity.

**Figure 2:**
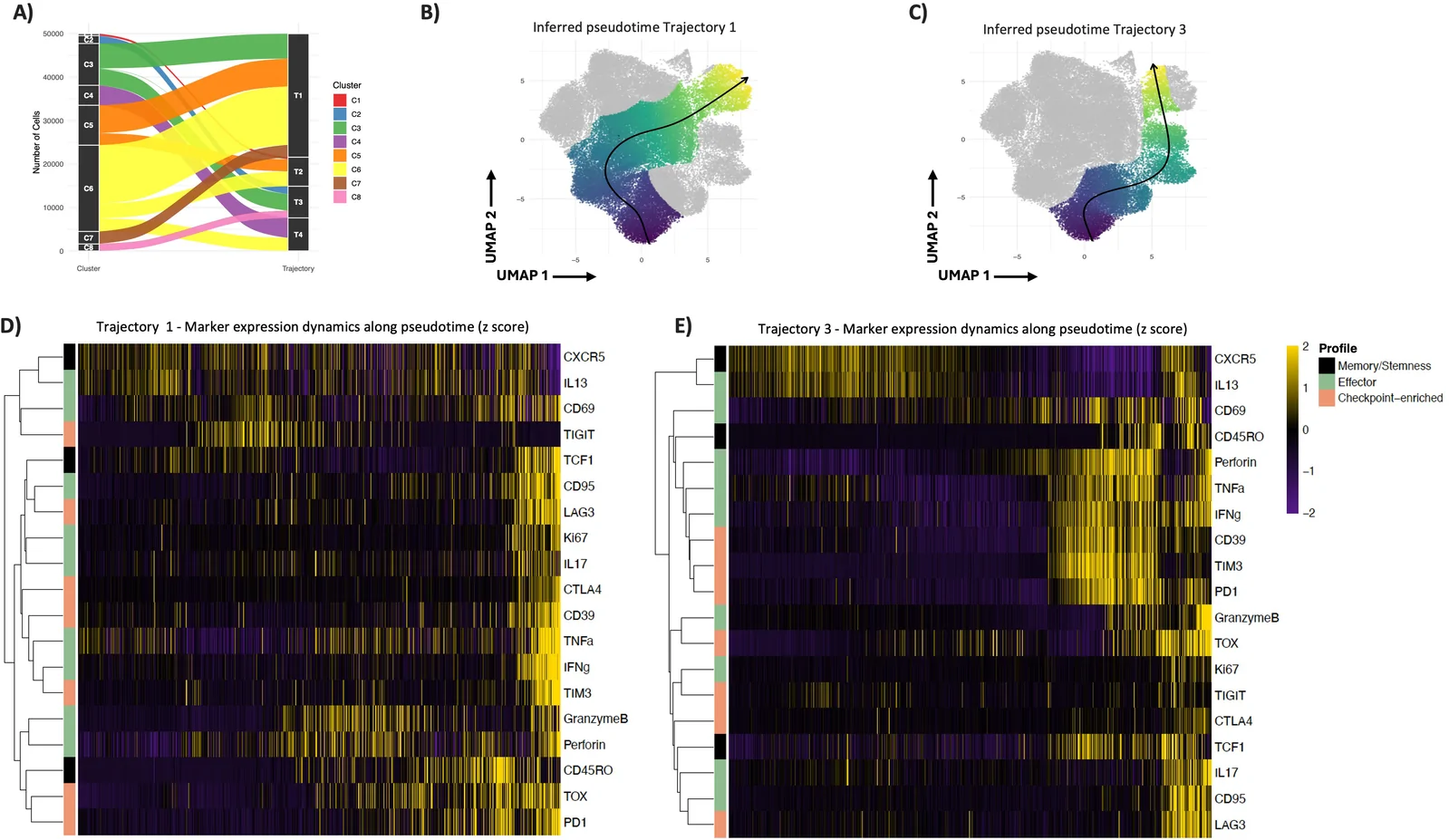
Pseudotime analysis identified inferred phenotypic trajectories associated with immune checkpoint receptor expression in CD8^+^ T cells from patients with SLE. Pseudotime inference was performed on CD8^+^ T cell clusters identified by unsupervised analysis. **(A)** Sankey diagram illustrates the relationship between CD8^+^ T cell clusters and inferred phenotypic trajectories. **(B, C)** Slingshot identified four inferred trajectories (T1-T4). The two principal trajectories (T1 and T3) are visualized on the UMAP embedding and colored according to pseudotime values. **(D, E)** Heatmap shows the z score-normalized expression of phenotypic and functional markers along trajectories T1 and T3. Expression of effector-associated and cytotoxic-associated markers remained detectable across pseudotime and was observed together with coordinated enrichment of immune checkpoint-associated markers. Cells are ordered according to inferred pseudotime values (left to right). Additional trajectories (T2 and T4) are shown in **Supplementary Figure 4**.

The CXCR5^low^ memory-like Cluster 3 was selected as the analytical reference because it displayed the lowest combined expression of effector molecules and immune checkpoint receptors, positioning it at one extreme of the phenotypic spectrum identified in our dataset. Importantly, this choice reflects an analytical reference rather than an assertion of a defined developmental origin. Trajectory inference identified four inferred trajectories (T1-T4), visualized through Sankey diagrams and UMAP projections (**Figure 2A**). These trajectories were organized around clusters characterized by coordinated immune checkpoint receptor expression together with expression of effector-associated molecules.

The main trajectories, T1 and T3, diverged toward distinct phenotypic endpoints. Trajectory T1 was inferred to connect Cluster 5 with Cluster 7, the multicheckpoint-enriched population. In contrast, trajectory T3 extended toward Cluster 8, passed through Cluster 2 and ultimately converged on Cluster 7. Along the inferred trajectories, we observed coordinated enrichment of immune checkpoint-associated markers (PD-1, TOX, CD39, LAG-3 and TIM-3) occurring in parallel with expression of effector-associated molecules, including Granzyme B, Perforin, TNFα and IFNγ. These observations suggest that coordinated enrichment of immune checkpoint-associated markers coexists with expression of effector-associated molecules across the inferred phenotypic trajectories (**Figures 2B, C**). Trajectory T2 terminated in Cluster 1, which was characterized by high expression of Perforin and Ki-67 and was predominantly expanded in aSLE, consistent with an actively proliferating cytotoxic subset. In contrast, trajectory T4 terminated in Cluster 4, defined by the expression of CXCR5, CD69, IL-13 and IL-17A, representing an immunophenotypically distinct CD8^+^ T cell state from the cytotoxic-associated phenotypes identified in the other trajectories. The relative stability of Cluster 4 across disease groups indicated that this subset was maintained independent of disease activity (**Supplementary Figures 4A, B**).

Collectively, these findings suggest that CD8^+^ T cell populations in SLE are distributed along inferred phenotypic trajectories characterized by coordinated immune checkpoint-associated marker enrichment together with retained expression of effector-associated molecules. These inferred trajectories support a continuum of activation states in which checkpoint-associated marker enrichment coexists with sustained expression of effector-associated molecules.

To further characterize marker dynamics along each trajectory, marker expression was normalized using z scores and visualized across pseudotime as heatmaps. This analysis revealed enrichment of immune checkpoint receptors, including PD-1, TIM-3 and CD39, together with the transcriptional regulator TOX, which occurred in parallel with the sustained expression of effector-associated molecules such as Granzyme B, Perforin, TNFα and IFNγ. Visualization of marker expression across pseudotime further highlighted distinct trajectory-associated expression patterns, with T1 and T3 displaying tightly coordinated immune checkpoint-associated marker and effector-associated molecule coexpression (**Figures 2D, E**; **Supplementary Figures 4C, D**).

### 3.3 Chronic TCR/CD28 stimulation induces immune checkpoint receptor acquisition while retaining effector-associated features in CD8^+^ T cells

**Figure 3:**
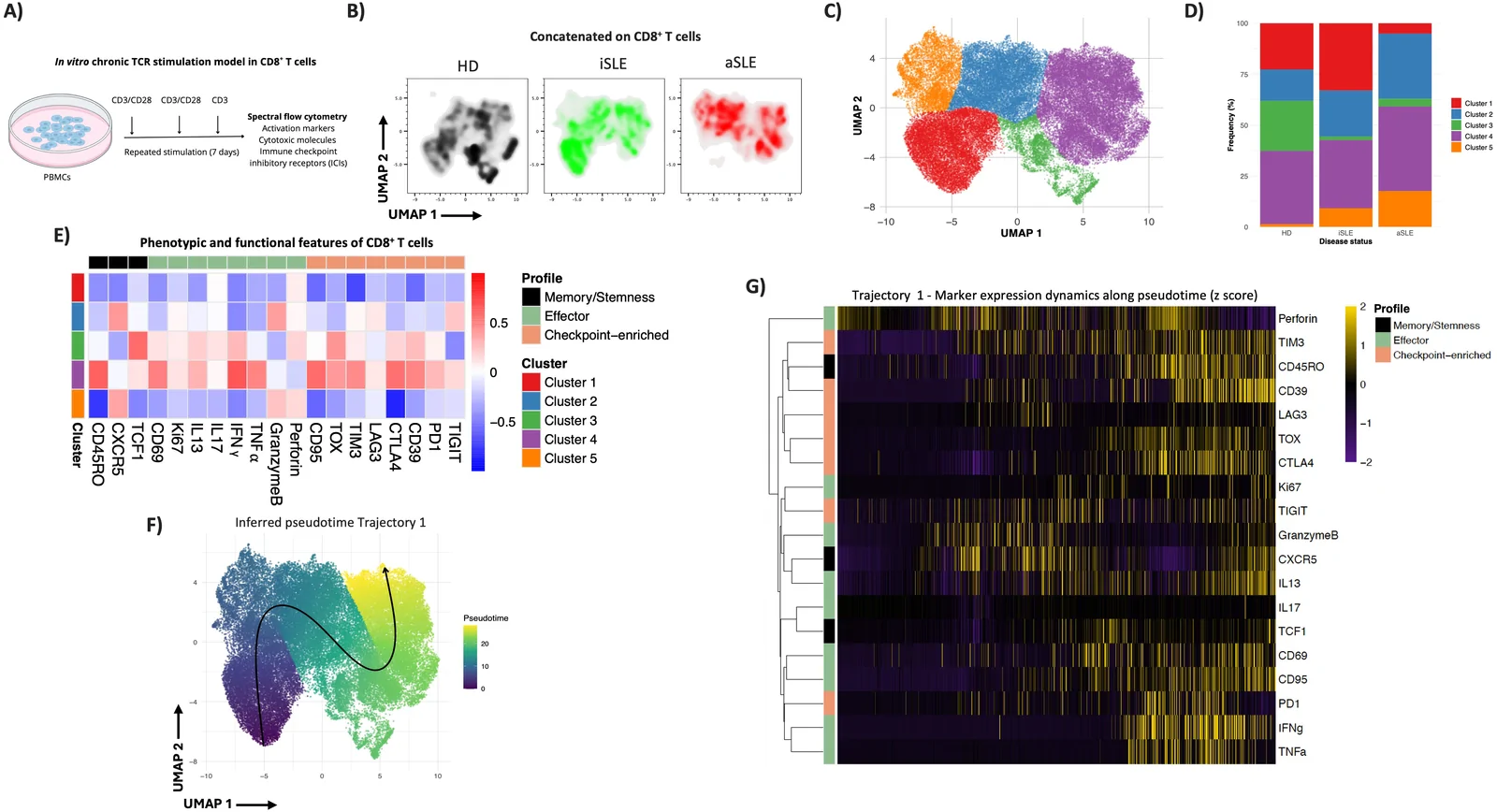
*In vitro* chronic TCR/CD28 stimulation induces immune checkpoint receptor acquisition while retaining effector-associated marker expression in CD8^+^ T cells. **(A)** Peripheral blood mononuclear cells (PBMCs) from healthy donors (HD; n=12), patients with inactive SLE (iSLE; n=36), and patients with active SLE (aSLE; n=19) were subjected to repeated *in vitro* stimulation with anti-CD3 and anti-CD28 antibodies to model sustained TCR signaling; the experimental design is shown. **(B)** UMAP projections of concatenated CD8^+^ T cells illustrate the global phenotypic landscape following chronic stimulation across disease groups. **(C)** Unsupervised clustering revealed five phenotypically distinct CD8^+^ T cell subsets visualized by UMAP embedding. **(D)** Relative distribution of CD8^+^ T cell clusters across disease states following chronic stimulation. **(E)** Heatmap of z score-normalized marker expression highlighting phenotypic and functional heterogeneity across the identified CD8^+^ T cell clusters. **(F)** Slingshot-based pseudotime inference of CD8^+^ T cell phenotypic states visualized on the UMAP embedding. **(G)** Heatmap of z score-normalized marker expression along pseudotime illustrates coordinated enrichment of immune checkpoint-associated markers together with retained effector-associated marker expression. Cells are ordered according to inferred pseudotime values (left to right).

To investigate whether sustained TCR engagement could contribute to the checkpoint-enriched phenotypes observed *ex vivo* in patients with SLE, we established an *in vitro* chronic stimulation model based on repeated CD3/CD28 activation. This model was designed to determine whether prolonged stimulation alone could reproduce key phenotypic features identified in circulating T cells, thereby complementing the *ex vivo* analyses. PBMCs were repeatedly stimulated with anti-CD3 and anti-CD28 antibodies (**Figure 3A**). Phenotypic profiles obtained after chronic *in vitro* stimulation were interpreted in the context of the *ex vivo* landscape, which served as the reference framework for comparison. Dimensionality reduction using UMAP revealed marked remodeling of the CD8^+^ T cell compartment upon chronic stimulation, characterized by redistribution of cells across phenotypic groups (**Figure 3B**). Expression patterns of individual markers included in the staining panel are shown as UMAP feature plots in **Supplementary Figure 5**.

Clustering analysis revealed five major CD8^+^ T cell clusters under *in vitro* stimulation conditions (**Figure 3C**). Cluster 1 showed a lower relative representation in patients with aSLE compared with patients with iSLE and HD, whereas iSLE samples exhibited a modest increase relative to HD. This cluster was characterized primarily by low Perforin expression, consistent with lower cytotoxic-associated marker expression. Cluster 2, defined by expression of CXCR5 and Granzyme B together with low levels of LAG-3 and TIGIT, showed a modestly higher relative representation in aSLE, consistent with a partially activated cytotoxic phenotype. In contrast, Cluster 3 showed a higher relative representation in HD-derived cultures and exhibited a complex activation-associated profile characterized by expression of multiple immune checkpoint receptors, including TIM-3, CTLA-4 and CD39, together with the transcriptional regulators TOX and TCF1, activation and proliferation-associated markers CD69 and Ki67, and effector-associated molecules including IL-13, IL-17A, IFNγ and Perforin, while it displayed low expression of CD45RO and PD-1. This phenotype is consistent with sustained activation accompanied by coordinated immune checkpoint receptor expression. Notably, despite relatively lower expression of selected cytotoxic-associated markers, Cluster 3 retained expression of cytokines, particularly IFNγ. These findings indicate that immune checkpoint receptor expression in this context coexists with detectable cytokine expression despite lower expression of selected cytotoxic-associated markers.

Cluster 4 also exhibited high expression of multiple immune checkpoint receptors. However, in contrast to Cluster 3, this population displayed lower expression of IL-17A and Perforin, accompanied by increased expression of CD95 and multiple immune checkpoint receptors, including TIM-3, LAG-3, CTLA-4, CD39 and TIGIT. This phenotype is consistent with a highly activated CD8^+^ T cell state characterized by extensive immune checkpoint receptor expression. Despite these phenotypic characteristics, the frequency of Cluster 4 did not vary significantly between groups. Notably, IFNγ expression was maintained, whereas TNFα expression was increased despite extensive immune checkpoint receptor expression. Finally, Cluster 5 showed a higher relative representation in patients with SLE, particularly in those with active disease, and was characterized by coexpression of CXCR5, Granzyme B and Perforin, representing a cytotoxic-associated CD8^+^ T cell phenotype preferentially expanded in patients with active disease (**Figures 3D, E**).

Together, these results suggest that chronic TCR/CD28 stimulation *in vitro* partially recapitulates immune checkpoint receptor acquisition observed *ex vivo* without complete loss of cytotoxic or cytokine-associated marker expression. To further contextualize these phenotypic transitions, we applied pseudotime inference to organize CD8^+^ T cell states according to their relative marker expression profiles. Cells were positioned based on relative expression gradients of activation, effector and inhibitory markers. This analysis revealed an inferred phenotypic trajectory characterized by coordinated enrichment of immune checkpoint-associated markers, including PD-1, TOX, LAG-3 and CD39, together with CD45RO enrichment. Along this continuum, expression of cytotoxic mediators such as Granzyme B and Perforin gradually decreased, whereas the expression of effector cytokines IFNγ and TNFα remained detectable (**Figures 3F, G**).

**Figure 4:**
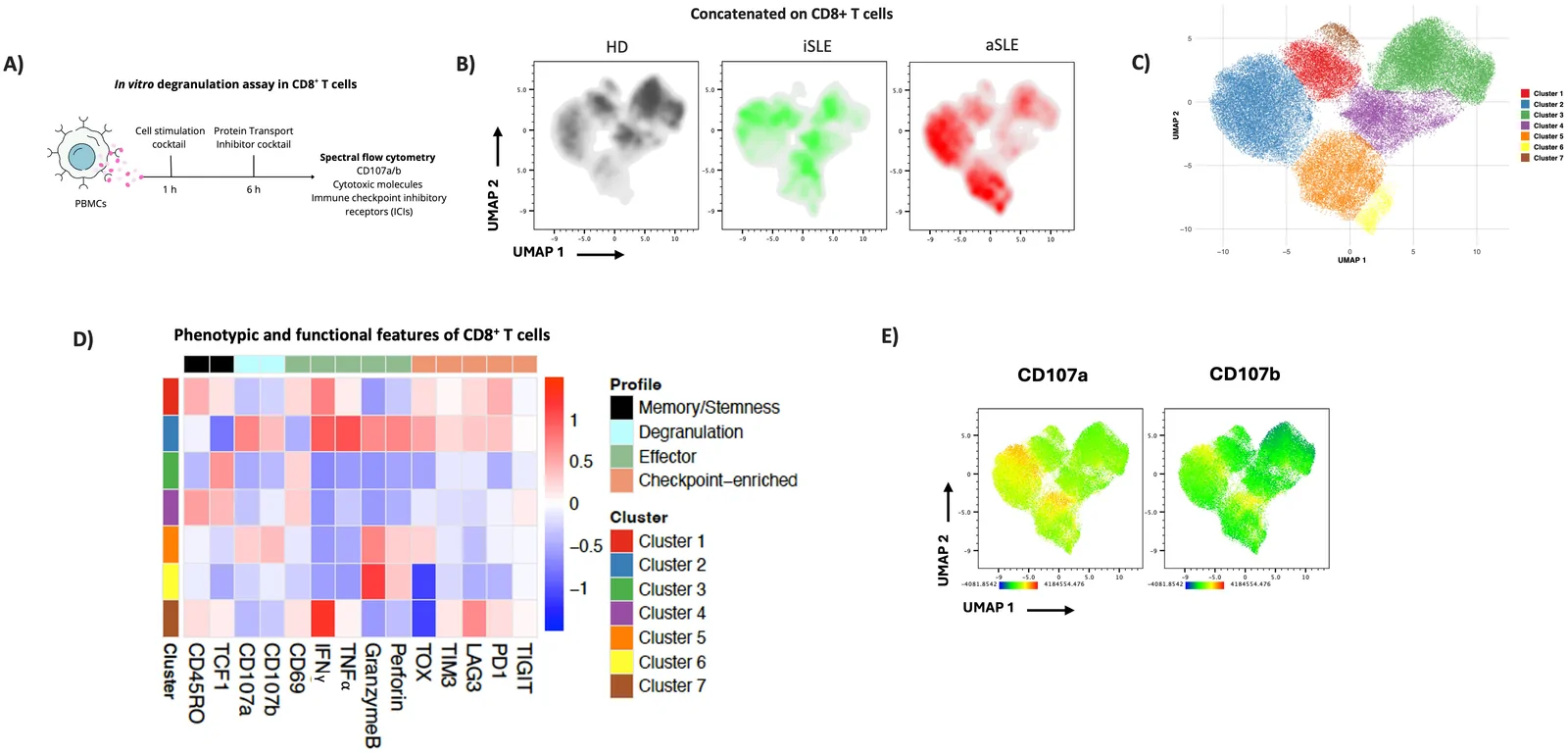
CD8^+^ T cells from patients with SLE retain expression of degranulation-associated markers despite immune checkpoint receptor expression. **(A)** Peripheral blood mononuclear cells (PBMCs) from healthy donors (HD; n=3), patients with inactive SLE (iSLE; n=3), and patients with active SLE (aSLE; n=3) were subjected to a short-term *in vitro* degranulation assay. Cells were stimulated with a cell stimulation cocktail for 1 h, followed by incubation with a protein transport inhibitor cocktail for 6 h prior to flow cytometric analysis; the experimental design is shown. **(B)** UMAP projections of concatenated CD8^+^ T cells illustrate the global phenotypic landscape across disease groups following degranulation stimulation. **(C)** Unsupervised clustering revealed seven phenotypically distinct CD8^+^ T cell subsets visualized by UMAP embedding. **(D)** Heatmap of z score-normalized marker expression highlights coordinated patterns of memory-associated, degranulation (CD107a/b), effector molecules and immune checkpoint receptors across CD8^+^ T cell clusters. **(E)** UMAP feature plots showing the expression of CD107a and CD107b across the CD8^+^ T cell landscape.

To determine whether immune checkpoint receptor expression coexisted with the expression of degranulation-associated and effector-associated markers, we evaluated CD107a (LAMP-1) and CD107b (LAMP-2) in CD8^+^ T cells. Expression patterns of individual markers included in the staining panel are shown as UMAP feature plots in **Supplementary Figure 6**. Unsupervised UMAP, clustering and heatmap analyses revealed detectable expression of degranulation-associated markers in both patients with SLE and HD, despite expression of multiple immune checkpoint receptors. These expression patterns were consistent with our *in vitro* chronic stimulation model, in which coordinated enrichment of checkpoint-associated markers occurred without a marked loss of effector cytokine expression. Collectively, these findings suggest that chronic TCR/CD28 stimulation reproduces key phenotypic features observed in CD8^+^ T cells from patients with SLE. In both the *ex vivo* and *in vitro* settings, CD8^+^ T cells exhibited detectable expression of effector-associated and degranulation-associated markers despite expression of immune checkpoint receptors, mirroring the phenotypic profiles observed in patients with SLE (**Figures 4A–E**).

### 3.4 Disease activity in SLE reshapes the CD4^+^ T cell compartment through expansion of multicheckpoint-enriched populations.

**Figure 5:**
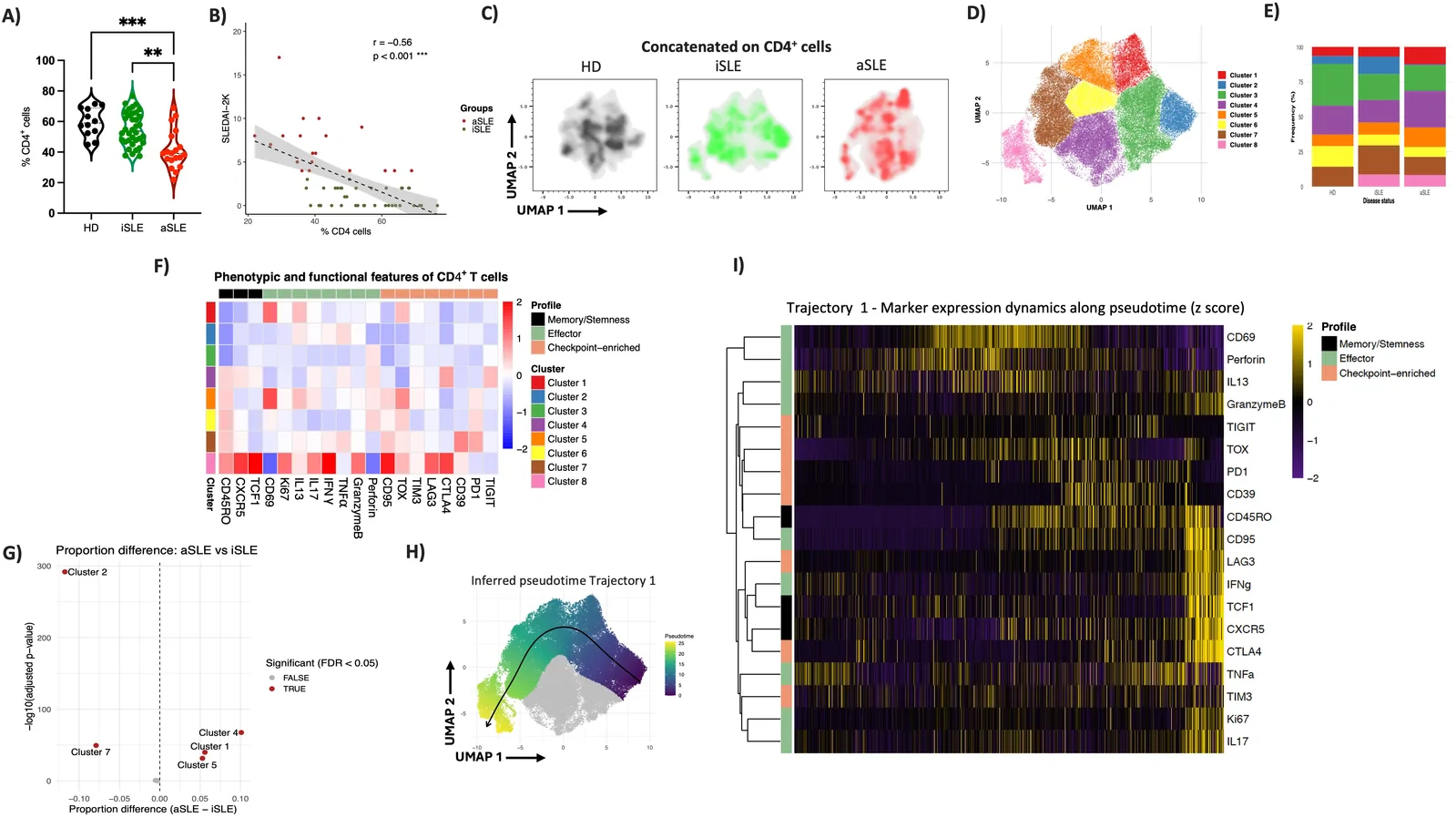
High-dimensional analysis revealed CD4^+^ T cell subset-enriched immune checkpoint receptor expression in patients with SLE. **(A)** Frequency of total CD4^+^ T cells in healthy donors (HD; n=12), patients with inactive SLE (iSLE; n=36), and patients with active SLE (aSLE; n=19). **(B)** Negative correlation between CD4^+^ T cell frequency and disease activity, as measured by the SLEDAI-2K score (n = 55). **(C)** UMAP projections of concatenated CD4^+^ T cells illustrate the global phenotypic landscape across disease states. **(D)** Unsupervised clustering revealed eight phenotypically distinct CD4^+^ T cell populations. **(E)** Relative frequencies of CD4^+^ T cell clusters across disease states. **(F)** Heatmap of z score-normalized marker expression highlighting the phenotypic and functional heterogeneity across CD4^+^ T cell clusters. **(G)** Differential cluster abundance between patients with aSLE and patients with iSLE shown as proportion differences with false discovery rate (FDR)-adjusted p values. **(H)** Pseudotime inference of CD4^+^ T cell phenotypic states shows the principal trajectory (T1) visualized on the UMAP embedding. An additional trajectory (T2) is shown in **Supplementary Figure 8**. **(I)** Heatmap of z score-normalized marker expression along pseudotime. Graphs display individual values with median lines. Statistical significance was assessed using the Kruskal-Wallis test followed by Dunn’s *post hoc* multiple comparisons test (*p < 0.05; **p < 0.01; ***p < 0.001).

Given that CD8^+^ T cells exhibited disease activity-dependent shifts toward multicheckpoint-enriched effector states, we next asked whether CD4^+^ T cells displayed analogous or distinct patterns of phenotypic reorganization in SLE. The proportion of CD4^+^ T cells was significantly lower in patients with aSLE (median: 39.10; Q1-Q3: 34.70-50.40) than in patients with both iSLE (median: 53.70; Q1-Q3: 47.03-65.08) and HD (median: 59; Q1-Q3: 52.75-68.83; **Figure 5A**). Moreover, CD4^+^ T cell frequency negatively correlated with disease activity, as measured by SLEDAI-2K score (r = -0.56, p < 0.001***; **Figure 5B**), suggesting that reduced CD4^+^ T cell abundance is associated with increased disease activity.

To further investigate CD4^+^ T cell heterogeneity, we performed unsupervised clustering, which revealed eight major phenotypically distinct populations (**Figures 5C, D**). The expression patterns of the individual markers included in the staining panel are shown as UMAP feature plots in **Supplementary Figure 7**. The characteristics of each cluster are summarized in **Figures 5E, F** and **Supplementary Figure 8A**.

Cluster 1, characterized by the expression of CD69, IL-13, and TOX, was predominantly expanded in patients with aSLE, suggesting an activated CD4^+^ T cell subset with Th2-associated features in the context of chronic inflammation. Cluster 2, defined by low levels of IL-13, IFNγ, and TNFα, was enriched in iSLE but markedly reduced in patients with aSLE. This subset may represent a population preferentially associated with inactive disease. Cluster 3, characterized by low Perforin expression, was enriched in HDs. In Cluster 4, the expression of CD45RO, CXCR5, TNFα, Perforin, CTLA-4, and TIGIT was slightly greater in patients with aSLE than in HDs, consistent with a population displaying increased expression of activation-associated markers together with coordinated immune checkpoint receptor expression. Cluster 5 displayed high expression of CD45RO, CD69, IL-13, IL-17A, Perforin, CD95, TOX and low PD-1 levels, and was partially increased in aSLE, suggesting an activated-effector subset with proinflammatory potential. Cluster 6, predominant in HDs, expressed CD45RO, Perforin, TOX, and PD-1, and was decreased in patients with SLE. Cluster 7, enriched in patients with iSLE, expressed CD45RO, TNFα, CD95, TOX, CD39, and PD-1, consistent with a memory subset characterized by coordinated immune checkpoint receptor expression. Finally, Cluster 8 was markedly increased in patients with SLE but absent in HDs. This cluster exhibited high expression of multiple immune checkpoint-associated markers, including CD95, TOX, LAG-3 and CTLA-4, together with CD45RO, CXCR5 and TCF1, while displaying low CD39 expression and lacking PD-1. Despite this checkpoint-enriched profile, Cluster 8 retained Ki-67, IL-13, IL-17A, IFNγ and Granzyme B expression. Together, these features define an activated, proliferative CD4^+^ T cell population displaying exhaustion-associated traits while retaining selected effector-associated features.

The relative representation of CD4^+^ T cell clusters differed across disease groups. Cluster 2 was relatively enriched in patients with iSLE compared with patients with aSLE, whereas Cluster 8 showed a higher relative representation in SLE and was absent in HDs (**Figure 5G**; **Supplementary Figures 8B, C**). Given the prominent representation of Cluster 8 and its complex phenotype in the global analysis, we performed a secondary unsupervised reclustering focusing specifically on this multicheckpoint-enriched population to better resolve its internal heterogeneity. This approach revealed substantial phenotypic diversity, identifying distinct subclusters that shared immune checkpoint receptor expression but differed in the expression of effector and inflammatory markers, including cytotoxic mediators and proinflammatory cytokines (**Supplementary Figures 8D, E**). Several of these subclusters showed a higher relative representation in aSLE (**Supplementary Figure 8F**). Notably, markers such as IL-17A, which appeared at the cluster level in the initial analysis, were restricted to specific subclusters upon reclustering, indicating that IL-17A expression was not a defining feature of the multicheckpoint population. Several subclusters retained effector-associated features despite high checkpoint expression, highlighting that multicheckpoint CD4^+^ T cells in SLE encompass a spectrum of phenotypic states rather than a uniform functional program.

Together, these findings indicate that disease activity in SLE is associated with altered representation of activated and multicheckpoint-expressing CD4^+^ subsets. To determine whether these phenotypic states represent distinct populations or continuous phenotypic relationships, we next performed trajectory analysis.

Trajectory analysis revealed two inferred phenotypic trajectories (**Figure 5H**; **Supplementary Figure 8G**). We selected Cluster 2 as the analytical reference (root) population for trajectory inference because of its similarity to HD and the absence of strong activation markers. Trajectory 1 terminated at Cluster 8 in the inferred phenotypic ordering, which was markedly increased in patients with SLE but not in HD. This cluster retained expression of multiple checkpoint-associated markers together with detectable IFNγ and TNFα expression but lacked Perforin, suggesting a checkpoint-enriched phenotype with retained cytokine-associated features. Trajectory 2 terminated at Cluster 3 in the inferred phenotypic ordering, characterized by Perforin expression and phenotypic features resembling those observed in HD. Notably, this pathway bypassed the clusters enriched in patients with SLE who exhibited multicheckpoint-enriched phenotypes, suggesting a distinct cytotoxic phenotype preferentially represented in healthy donors.

To further examine the phenotypic organization along each inferred trajectory, we analyzed the expression of phenotypic and functional markers across pseudotime for both pathways (**Figure 5I**; **Supplementary Figure 8H**). In pathway 1, corresponding to the route leading to Cluster 8, the inferred phenotypic trajectory was characterized by coordinated enrichment of immune checkpoint-associated markers such as TOX, LAG-3, CTLA-4 and CD95, together with CD45RO and TCF1, accompanied by detectable expression of IFNγ, TNFα and Granzyme B. This pattern is consistent with coordinated enrichment of checkpoint-associated markers while retaining detectable expression of selected effector-associated markers. In contrast, pathway 2, which converged toward Cluster 3, retained Perforin expression and low expression of TNFα, with limited induction of checkpoint-enriched markers, consistent with a cytotoxic-associated phenotype resembling that observed in healthy donors. Notably, TIGIT expression emerged selectively along this trajectory, consistent with coordinated enrichment of selected checkpoint-associated markers while retaining effector-associated characteristics. Taken together, these pseudotime dynamics describe two distinct phenotypic continua: one characterized by coordinated enrichment of multicheckpoint-associated markers while retaining selected effector-associated marker expression and another characterized by retention of cytotoxic features typical of the phenotype predominating in healthy donors.

**Figure 6:**
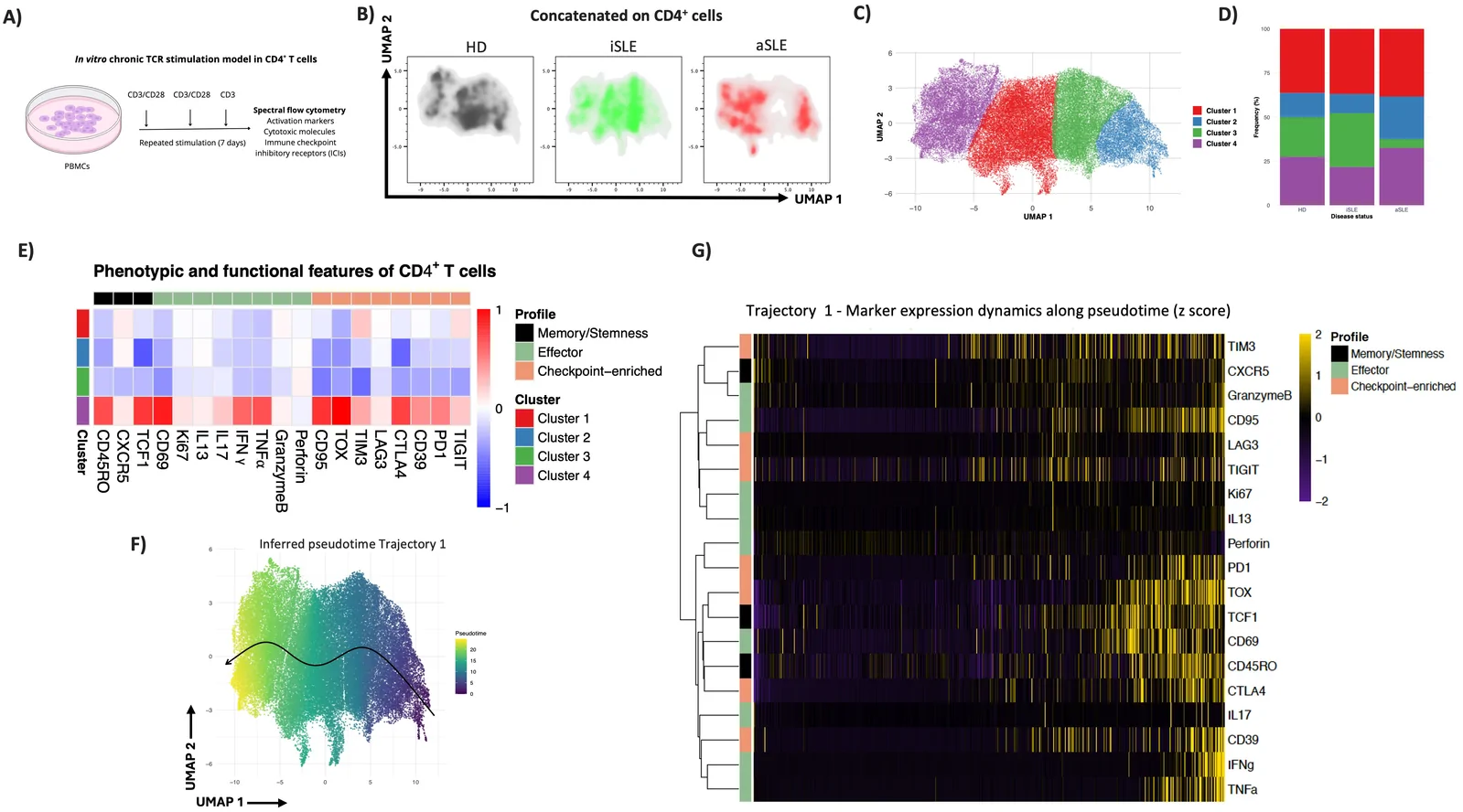
*In vitro* chronic stimulation of CD4^+^ T cells induces coordinated enrichment of immune checkpoint-associated markers together with retained expression of effector-associated markers. **(A)** Peripheral blood mononuclear cells (PBMCs) from healthy donors (HD; n=12), patients with inactive SLE (iSLE; n=36), and patients with active SLE (aSLE; n=19) were stimulated with anti-CD3 and anti-CD28 antibodies to model sustained TCR activation; the experimental design is shown. **(B)** UMAP projections of concatenated CD4^+^ T cells show distinct distribution patterns among groups following chronic stimulation. **(C)** Unsupervised clustering revealed four phenotypically distinct CD4^+^ T cell subsets. **(D)** Relative frequencies of CD4^+^ T cell clusters across groups. **(E)** Heatmap of z score-normalized marker expression highlighting phenotypic diversity across clusters. **(F)** Slingshot-based pseudotime inference identified a single inferred phenotypic trajectory. **(G)** Heatmap of z score-normalized marker expression along the inferred pseudotime trajectory. Cells are ordered according to inferred pseudotime values (left to right).

We next analyzed whether chronic stimulation in cell cultures promotes the acquisition of checkpoint-enriched phenotypes in CD4^+^ T cells, following the same experimental model used for CD8^+^ T cells. Unsupervised clustering and UMAP projection revealed that the majority of CD4^+^ T cells acquired a multicheckpoint phenotype upon chronic stimulation. The expression patterns of individual markers included in the staining panel are shown as UMAP feature plots in **Supplementary Figure 9**. Cluster 1 was characterized by the expression of CXCR5, TIM-3, and TIGIT, consistent with an activated but checkpoint-modulated phenotype. In contrast, Clusters 2 and 3 displayed minimal expression of checkpoint-enriched markers, suggesting populations with limited checkpoint receptor expression. Based on these features, Cluster 2 was selected as the analytical reference population for trajectory inference. Cluster 4 displayed broad expression of checkpoint-enriched markers while lacking Perforin, indicating an activated phenotype (**Figures 6A–G**). To further evaluate whether checkpoint-enriched CD4^+^ T cells retained effector-associated features, we performed an *in vitro* degranulation assay. Unsupervised analysis identified heterogeneous CD4^+^ T cell populations with distinct expression patterns of degranulation-associated molecules, cytotoxic mediators, cytokines, and immune checkpoint receptors (**Supplementary Figure 10**). Taken together, these *in vitro* analyses indicate the coordinated induction of multiple checkpoint-enriched markers in CD4^+^ T cells while retaining effector-associated features.

### 3.5 Linear discriminant analysis integrating CD8^+^ T cell phenotypes in SLE

Linear discriminant analysis (LDA) was performed using ten selected *ex vivo* CD8^+^ T cell immunological variables derived from the supervised analyses (**Supplementary Table 3**). LDA was applied as an integrative dimensionality-reduction approach to summarize coordinated immunophenotypic variation across individuals.

**Figure 7:**
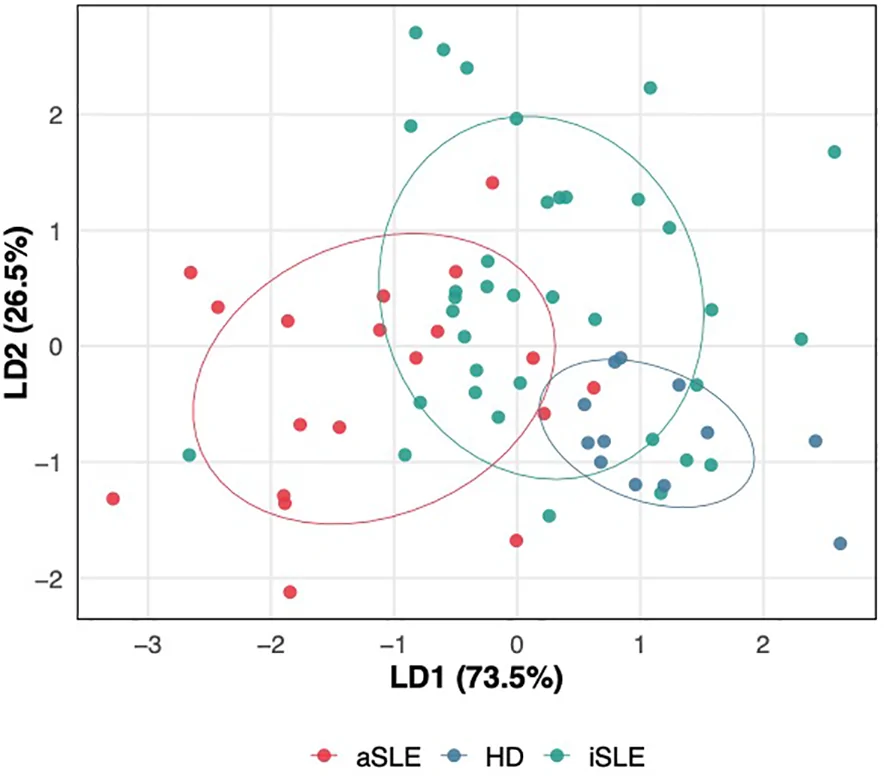
Distinct immunological signatures characterize active and inactive SLE. Linear discriminant analysis (LDA) was performed using ten donor-level immunological variables derived from the *ex vivo* CD8^+^ T cell analyses (**Supplementary Table 3**). Each point represents one individual. LDA was used as a dimensionality-reduction approach to visualize group separation. The first two discriminant functions explained 73.5% (LD1) and 26.5% (LD2) of the discriminant variance and showed partial separation among healthy donors (HD), inactive SLE (iSLE) and active SLE (aSLE).

The resulting discriminant functions generated a dominant first axis (LD1, 73.5% of the explained variance) that distinguished the distributions of HD, iSLE, and aSLE samples, whereas LD2 accounted for 26.5% of the explained variance (**Figure 7**). HD samples showed a relatively compact distribution in the LDA space, consistent with the predominance of homeostatic CD8^+^ T cell immunophenotypes characterized by low inhibitory receptor expression. In contrast, iSLE samples occupied an intermediate and more heterogeneous position, whereas aSLE samples were shifted predominantly toward negative LD1 values. Although partial overlap was observed between inactive and active SLE, the distribution of samples reflected changes in the integrated CD8^+^ T cell immunological signatures across the study groups. This pattern paralleled the immunophenotypic signatures identified in the preceding supervised analyses, including proliferative, cytotoxic and multicheckpoint-enriched CD8^+^ T cell phenotypes.

To further evaluate the relationship between the integrated immunological signature and continuous disease activity, the first discriminant axis (LD1), generated from the ten immunological variables included in the LDA model, was correlated with continuous SLEDAI-2K scores. LD1 was significantly associated with SLEDAI-2K scores (Spearman ρ = −0.57, p < 0.001), supporting an association between the integrated immunological signature and clinical disease activity (**Supplementary Figure 11**).

## 4 Discussion

Immune checkpoint receptors are widely used as markers of T cell exhaustion and dysfunction in chronic infections and cancer (32–34). However, their interpretation in autoimmune disease remains unclear. In systemic lupus erythematosus (SLE), immune checkpoint-expressing CD4^+^ and CD8^+^ T cells have been reported across disease states, yet whether these phenotypes represent terminal exhaustion, distinct phenotypic states, or persistent activation remains unresolved. Clarifying the phenotypic significance and functional implications of immune checkpoint receptor expression in SLE is therefore critical, as it has direct implications for disease classification, biomarker interpretation and therapeutic targeting.

In this study, we performed a high-dimensional characterization of circulating CD4^+^ and CD8^+^ T cell states in patients with active and inactive SLE, integrating *ex vivo* profiling, *in vitro* models of sustained TCR/CD28 stimulation, and trajectory-based analyses. Across these complementary approaches, we consistently identified T cell populations characterized by coordinated expression of multiple immune checkpoint inhibitory receptors that nonetheless retained expression of cytotoxic-associated markers and cytokines. These observations reveal a complex relationship between immune checkpoint receptor expression and T cell functional state in SLE, suggesting that exhaustion-associated signatures in this context may not uniformly correspond to terminal functional impairment, but rather reflect alternative, checkpoint-enriched activation states.

Indeed, although expression of inhibitory receptors such as PD-1, TIM-3, LAG-3, TIGIT, and CD39 is commonly interpreted as evidence of exhaustion (35, 36), our analyses indicate that increased immune checkpoint receptor expression in SLE does not necessarily equate to functional inhibition. Multicheckpoint-enriched populations, particularly within the CD8^+^ T cell compartment, retain expression of Perforin, Granzyme B and selected effector cytokines, including IFNγ and TNFα. Moreover, the enrichment of cytotoxic, proliferative, and highly checkpoint-enriched clusters in patients with active disease underscores the persistence of effector-associated features despite extensive inhibitory receptor engagement. Together, these findings indicate that immune checkpoint receptor expression in SLE does not necessarily mark loss of effector-associated features but instead accompanies distinct activation-associated states that emerge under chronic immune activation.

These results suggest that, rather than reflecting a uniform transition toward terminal exhaustion, increased immune checkpoint receptor expression in SLE occurs in the context of sustained inflammatory signaling and continuous helper-dependent cues. In autoimmune settings, persistent CD4^+^ T cell-B cell interactions, together with chronic exposure to inflammatory cytokines and autoreactive stimulation, may influence how inhibitory pathways modulate T cell differentiation and function (12, 13). In line with this interpretation, studies in a type 1 diabetes model have shown that autoreactive CD4^+^ T cells can maintain TCF1 expression under persistent antigen exposure, which is associated with sustained survival and functional capacity in chronic autoimmunity (37). Under these conditions, a helper-rich inflammatory milieu may favor alternative transcriptional and functional programs that are compatible with retained effector-associated characteristics despite extensive immune checkpoint receptor expression, although the epigenetic and metabolic adaptations to chronic activation remain to be established.

Beyond mechanistic insights from experimental autoimmune models, similar checkpoint-enriched yet functionally active T cell states have also been observed in human chronic inflammatory diseases. Consistent with this broader framework, exhaustion-like phenotypes have been associated with improved clinical outcomes in some autoimmune contexts, as they are more frequently observed in patients with disease remission (18). However, our results, together with findings from other chronic inflammatory diseases, indicate that checkpoint-expressing T cells do not uniformly exhibit phenotypes consistent with terminal exhaustion. Similar phenomena have been described in axial spondyloarthritis, including ankylosing spondylitis. Although traditionally classified as an autoimmune disease, it is now recognized as a prototypical autoinflammatory disorder driven primarily by dysregulation of the IL-17/IL-23 axis (38, 39). In this setting, CD127^-^ PD-1^+^ TIGIT^+^ CD8^+^ T cell subsets within synovial fluid retain robust effector activity, including high expression of IFNγ, TNFα, Granzyme B and Perforin, contributing to sustained inflammation and joint damage (40).

Notably, Tang et al. (2025) further reported reduced TOX expression in synovial CD8^+^ CD127^–^ PD-1^+^ TIGIT^+^ T cells compared with peripheral blood, indicating that acquisition of inhibitory receptors does not necessarily culminate in terminal exhaustion within inflamed tissues (40). Moreover, the relative reduction of CD8^+^ CXCR5^+^ T cells observed in peripheral blood from patients with active SLE may reflect redistribution toward secondary lymphoid tissues, consistent with the CXCR5–CXCL13 axis described in chronic inflammatory states (41).

Our pseudotime analyses further support this model by identifying inferred phenotypic trajectories in which CD8^+^ T cells exhibit coordinated enrichment of checkpoint-associated markers without converging on a phenotype consistent with terminal exhaustion. Instead, trajectory inference positioned cells along checkpoint-enriched phenotypic states characterized by TOX, TCF1 and multiple inhibitory receptors while maintaining expression of cytotoxic mediators and inflammatory cytokines.

This pattern is consistent with a checkpoint-enriched activation state, in which inflammatory signals and continuous co-stimulation, particularly via CD28, may contribute to limiting the emergence of phenotypes consistent with terminal exhaustion. Supporting this notion, chronic *in vitro* TCR/CD28 stimulation did not induce complete loss of cytotoxic-associated marker expression. The persistence of this state may be reinforced by the inflammatory milieu characteristic of SLE. Continuous CD28 costimulation (42), together with IL-21 and type I interferon signaling (43), may sustain effector-associated phenotypes and prevent the establishment of phenotypes consistent with terminal exhaustion. In chronic settings, persistent CD28 engagement is known to activate metabolic and survival pathways, including PI3K-AKT-mTOR signaling, which support the energetic demands of prolonged T cell activation (44).

Consistent with this, Humblin et al. (2023) demonstrated that sustained strong CD28 signaling in CD8^+^ T cells increases IRF4 expression and enhances glycolytic metabolism, facilitating the progression of a fraction of the TCF-1^+^ precursor populations toward CD39^+^ subsets without enforcing terminal exhaustion (42). Together, these mechanisms support a model in which checkpoint-enriched marker expression coexists with, rather than necessarily indicating suppression of, effector-associated phenotypes in SLE.

The identification of multicheckpoint-enriched effector states with retained cytotoxic-associated features has important implications for biomarker interpretation and therapeutic strategies. Checkpoint expression alone may be insufficient to define disease quiescence, and combined phenotypic signatures may more accurately stratify patients according to inflammatory burden or flare risk. Moreover, the persistence of cytotoxic and inflammatory T cells despite inhibitory receptor expression suggests that these populations may serve as sustained sources of tissue-damaging mediators, perpetuating chronic inflammation.

Because multicheckpoint-enriched T cell states in SLE do not necessarily correspond to loss of effector-associated characteristics, checkpoint molecules should not be interpreted as unequivocal markers of functional exhaustion in SLE. These observations underscore the need for caution when considering therapeutic strategies aimed at reversing exhaustion, such as immune checkpoint blockade in lupus. Instead, therapeutic approaches that reduce antigenic load, modulate co-stimulatory pathways, or dampen inflammatory cytokine signaling may represent alternative strategies to restore immune balance.

Altogether, our findings are consistent with a model in which chronic autoimmunity fosters activation states characterized by extensive immune checkpoint receptor expression alongside preserved effector-associated features. Rather than reflecting terminal exhaustion, these states likely represent dynamically regulated T cell phenotypes shaped by persistent inflammatory cues and sustained co-stimulatory signaling. This framework refines how immune checkpoint-associated T cell states are interpreted in SLE and underscores the need to consider activation, regulation, and functional plasticity when evaluating T cell dysfunction in systemic autoimmunity.

## 5 Study limitations

This study has several limitations that should be considered. The relatively modest cohort size warrants validation in larger, independent populations to confirm broader clinical applicability. Additionally, analyses were restricted to peripheral blood, and therefore tissue-specific immune dynamics could not be directly assessed. Future studies incorporating tissue samples and longitudinal follow-up will help define the stability, distribution and temporal dynamics of checkpoint-enriched T cell states in SLE.

## Data Availability

The original contributions presented in the study are included in the article/**Supplementary Material**. Further inquiries can be directed to the corresponding author.
