# Supplementary material for: Checkpoint-enriched T cells with retained effector-associated features are associated with disease activity in systemic lupus erythematosus
Source: Front Immunol. 2026 Sep 1;17:1818795. doi: 10.3389/fimmu.2026.1818795 (PMC13575747; doi:10.3389/fimmu.2026.1818795)
Supplement: Supplementary file 1 [file SupplementaryFile1.docx]

Supplementary Material

# 1 Supplementary Figures and Tables

Supplementary Table S1

| **Antibody or Reagent** | **Clone** | **Source** |
| --- | --- | --- |
| **Surface antibodies** |  |  |
| BV650 anti-human CD3 | OKT3 | BioLegend |
| PE/Fire810 anti-human CD4 | SK3 | BioLegend |
| APC/Fire810 anti-human CD8 | SK1 | BioLegend |
| BV510 anti-human CD69 | FN50 | BioLegend |
| PE/Fire700 anti-human CXCR5 (CD185) | J252D4 | BioLegend |
| BV570 anti-human CD45RO | UCHL1 | BioLegend |
| Spark Red 718 anti-human PD-1 (CD279) | A17188B | BioLegend |
| APC anti-human TIM-3 (CD366) | A18087E | BioLegend |
| Spark NIR 685 anti-human CD39 | A1 | BioLegend |
| PE anti-human CTLA-4 (CD152) | BNI3 | BioLegend |
| BV785 anti-human LAG-3 (CD223) | 11C3C65 | BioLegend |
| BV605 anti-human CD95 (Fas) | DX2 | BioLegend |
| BV480 anti-human TIGIT | 741182 | BD Biosciences |
| Alexa Fluor 488 anti-human CD107a (LAMP-1) | H4A3 | BioLegend |
| PE anti-human CD107b (LAMP-2) | H4B4 | BioLegend |
| **Intracellular antibodies** |  |  |
| PE/Dazzle 594 anti-human IL-17A | BL168 | BioLegend |
| Pacific Blue anti-human TNF-α | MAb11 | BioLegend |
| APC/Fire 750 anti-human IFN-γ | 4S.B3 | BioLegend |
| PerCP anti-human Granzyme B | QA18A28 | BioLegend |
| PerCP/Cyanine5.5 anti-human Perforin | dG9 | BioLegend |
| BV750 anti-human Ki-67 | Ki-67 | BioLegend |
| PE/Fire640 anti-human TCF1 (TCF7) | 7F11A10 | BioLegend |
| BV711 anti-human IL-13 | JES10-5A2 | BioLegend |
| Alexa Fluor 488 anti-human Tox/Tox2  eFluor 660 anti-human Tox | E6G50  TXRX10 | Cell Signaling TECHNOLOGY  Invitrogen |
| **Viability dye** |  |  |
| Live or Dye 568/583 | *NA* | Biotium |
|  |  |  |
| Abbreviation: NA, not applicable. |  |  |
|  |  |  |

Supplementary Table S2

| **Cluster** | **Key markers** | **Annotation** |
| --- | --- | --- |
| C1 | Ki67, Perforin | Proliferative cytotoxic |
| C2 | IFNγ, TNFα, Perforin, TIM-3, TCF1, CD39, PD-1 | Early checkpoint enriched effector |
| C3 | CXCR5^low^ | Memory-like |
| C4 | CXCR5, CD69, IL-13, IL-17 ^low^ | Follicular associated |
| C5 | CD45RO, PD-1, TOX, CD95 | PD-1/TOX-associated memory |
| C6 | Granzyme B, Perforin, TIGIT | Cytotoxic TIGIT-associated |
| C7 | CD95, TOX, TIM-3, LAG-3, CTLA4, TCF1, CD39,  PD-1 + effector molecules | Late checkpoint enriched effector |
| C8 | TIM-3, PD-1, CD39, TCF1, IFNγ, TNFα, Perforin | Checkpoint-enriched effector |

Supplementary Table S3

| **Coefficients of the immunological variables included in the final linear discriminant analysis (LDA).** | **LD1** | **LD2** |
| --- | --- | --- |
| % CD8^+^ Ki67^+^ Perforin^+^ | 0.532 | 0.239 |
| MFI Perforin (CD8^+^ Ki67^+^ Perforin^+^) | -1.292 | -1.099 |
| MFI Ki67 (CD8^+^ Ki67^+^ Perforin^+^) | -1.263 | -0.728 |
| % CD8^+^ TIGIT^+^ | -0.287 | 0.794 |
| MFI Perforin (CD8^+^ TIGIT^+^ Granzyme B^+^ Perforin^+^) | 0.622 | -0.431 |
| MFI Granzyme B (CD8^+^ TIGIT^+^ Granzyme B^+^ Perforin^+^) | 0.313 | 0.762 |
| % CD8^+^ CD45RO^+^ PD-1^+^ TIM-3^+^ TOX^+^ | 0.708 | 0.390 |
| % CD8^+^ CD45RO^+^ PD-1^+^ TIM-3^+^ TOX^+^ Granzyme B^+^ Perforin^+^ | -1.123 | 0.380 |
| MFI Granzyme B (% CD8^+^ CD45RO^+^ PD-1^+^ TIM-3^+^ TOX^+^ Granzyme B^+^ Perforin^+^) | -0.325 | -0.188 |
| MFI Perforin (% CD8^+^ CD45RO^+^ PD-1^+^ TIM-3^+^ TOX^+^ Granzyme B^+^ Perforin^+^) | 0.849 | 1.120 |

Positive and negative coefficients indicate the direction of the association of each variable with the corresponding discriminant function (LD1 and LD2).

| **Demographic and treatment characteristics of the SLE cohort.** | **SLE** | **iSLE** | **aSLE** |
| --- | --- | --- | --- |
| Total n | 55 | 36 | 19 |
| Female | 46 (83%) | 30 (83%) | 16 (84%) |
| Age, years | 36 (28–46) | 36 (30–44) | 34 (28–51) |
| Treatments |  |  |  |
| Corticosteroids | 21 (38.2%) | 9 (25.0%) | 12 (63.1%) |
| Antimalarials | 36 (65.4%) | 24 (66.6%) | 12 (63.1%) |
| Mycophenolate | 41 (74.5%) | 25 (69.4%) | 16 (84.2%) |
| Azathioprine | 9 (16.4%) | 7 (19.4%) | 2 (10.5%) |
| Rituximab | 1 (1.8%) | 1 (2.7%) | 0 (0%) |
| Other ^f^ | 10 (18.8%) | 7 (19.4%) | 3 (15.7%) |

Supplementary Table S4

Data are presented as *n* (%) or median (interquartile range), as appropriate. SLE, systemic lupus erythematosus; iSLE, inactive systemic lupus erythematosus; aSLE, active systemic lupus erythematosus. ᶠ Includes tacrolimus, methotrexate, cyclophosphamide, and cyclosporine. The SLE cohort and treatment information have been previously reported [22].

## 1.1 Supplementary Figures

**Supplementary Figure S1. Gating strategy and representative fluorescence-minus-one (FMO) controls.** (A) Peripheral blood mononuclear cells (PBMCs) from healthy donors (HD; n=12), inactive SLE (iSLE; n=36), and active SLE (aSLE; n=19) were stained with antibodies against CD3, CD4, CD8, and the markers listed in Supplementary Table S1. Singlets were identified, lymphocytes were gated, and dead cells were excluded using a viability dye before gating on CD3⁺ T cells and subsequent identification of CD4⁺ and CD8⁺ T cell subsets. (B) Representative histograms showing fluorescence-minus-one (FMO) controls (gray) overlaid with stained samples (red) used to define positive gates for each marker.

**Supplementary Figure 2. UMAP visualization of CD8^+^ T cell subsets in patients with SLE.** (B-D) UMAP feature plots show the expression patterns of individual markers included in the CD8^+^ T cell staining panel, grouped according to functional categories: memory-associated (B), effector (C) and immune checkpoint-enriched markers (D).

**Supplementary Figure 3. Phenotypic validation of *ex vivo* CD8^+^ T cell subsets in patients with SLE.** (A) Chord diagram shows the association between CD8^+^ T cell clusters identified by unsupervised analysis and the expression of phenotypic and functional markers. (B) Volcano plots show differential cluster abundance between patients with SLE and HD. Clusters with significant differences after false discovery rate (FDR) correction (FDR < 0.05) are highlighted as red dots. (C) Representative plots and quantitative analysis of the frequency and median fluorescence intensity (MFI) of CD8⁺ Perforin^+^ Ki67⁺ T cells across disease groups. (D) Frequencies and MFI of CD8⁺ TIGIT^+^ T cells across groups. (E) Representative plots and frequencies of CD8⁺ CD45RO^-^ PD-1⁺ and CD8⁺ CD45RO^+^ PD-1⁺ T cell subsets. (F) Representative plots and frequencies of CD8⁺ CD45RO^+^ PD-1⁺ TIM-3^+^ TOX⁺ T cells.

**Supplementary Figure 4. Pseudotime trajectory analysis of CD8⁺ T cell differentiation states in SLE.** (A, B) UMAP-based pseudotime trajectories corresponding to inferred trajectories T2 (A) and T4 (B) in ex vivo CD8⁺ T cells. (D, E) Heatmaps show z score-normalized marker expression of phenotypic and functional markers along trajectories T2 (C) and T4 (D). Cells are ordered from early to late pseudotime (left to right).

**Supplementary Figure 5. UMAP visualization of chronically stimulated *in vitro* CD8^+^ T cells in SLE.** (A-C) UMAP feature plots generated from concatenated *in vitro*-stimulated CD8^+^ T cell datasets show the expression patterns of individual markers included in the staining panel. Markers are grouped according to functional categories: memory-associated markers (A), immune checkpoint-enriched markers (B) and effector markers (C). (D) Chord diagram shows the association between CD8^+^ T cell clusters identified under *in vitro* stimulation conditions and the expression of phenotypic and functional markers.

**Supplementary Figure 6. UMAP visualization of the degranulation profile of CD8^+^ T cells in SLE.** UMAP feature plots generated from concatenated stimulated CD8^+^ T cell datasets illustrate the expression patterns of individual markers associated with activation, cytotoxic effector function and immune checkpoint-enriched markers included in the staining panel.

**Supplementary Figure 7. UMAP visualization of *ex vivo* CD4^+^ T cells in SLE.** (A-C) UMAP feature plots generated from concatenated *ex vivo* CD4^+^ T cell datasets show the expression patterns of individual markers included in the staining panel. Markers are grouped according to functional categories: memory-associated markers (A), immune checkpoint-enriched markers (B) and effector markers (C).

**Supplementary Figure 8. Phenotypic validation of *ex vivo* CD4^+^ T cell subsets in patients with SLE.** (A) Chord diagram shows the association between CD4^+^ T cell clusters identified by unsupervised analysis and the expression of phenotypic and functional markers. (B, C) Volcano plots show differential cluster abundance in patients with aSLE or iSLE compared with HD. Clusters with significant differences after false discovery rate (FDR) correction (FDR < 0.05) are highlighted as red dots. (D) Secondary unsupervised reclustering of Cluster 8 in iSLE and aSLE samples visualized by UMAP projection. (E) Heatmap shows z score-normalized expression of phenotypic and functional markers across Cluster 8 subclusters identified by secondary reclustering. (F) Dot plots of relative frequency of Cluster 8 subclusters in iSLE and aSLE. (G) UMAP-based pseudotime trajectory corresponding to inferred trajectory T2 in *ex vivo* CD4⁺ T cells. (H) Heatmap shows z score-normalized expression dynamics of phenotypic and functional markers along trajectory T2. Cells are ordered from early to late pseudotime (left to right).

**Supplementary Figure 9. UMAP visualization of *in vitro* CD4^+^ T cells in SLE.** (A-C) UMAP feature plots generated from concatenated *in vitro* CD4^+^ T cell datasets show the expression patterns of individual markers included in the staining panel. Markers are grouped according to functional categories: memory-associated markers (A), immune checkpoint-enriched markers (B) and effector markers (C).

**Supplementary Figure 10. UMAP visualization of the degranulation profile of CD4^+^ T cells in SLE.** (A) Peripheral blood mononuclear cells from healthy donors (HD; n=3), patients with inactive SLE (iSLE; n=3), and patients with active SLE (aSLE; n=3) were subjected to a short-term *in vitro* degranulation assay. Cells were stimulated with a cell stimulation cocktail for 1 h, followed by incubation with a protein transport inhibitor cocktail for 6 h prior to flow cytometric analysis. (B) UMAP feature plots generated from concatenated stimulated CD4^+^ T cell datasets across groups. (C) Unsupervised clustering revealed eight phenotypically distinct CD4⁺ T cell clusters visualized by UMAP embedding. (D) Heatmap of z score-normalized marker expression highlights coordinated patterns of memory, degranulation (CD107a/b), effector molecules and immune checkpoint receptors across clusters. (E) UMAP feature plots generated from concatenated stimulated CD4^+^ T cell datasets illustrate the expression patterns of individual markers included in the staining panel.

**Supplementary Figure 11. Integrated immunological signature correlates with continuous disease activity in patients with SLE.** The first discriminant axis (LD1), generated from the ten immunological variables included in the LDA model, was evaluated against continuous SLEDAI-2K scores in patients with SLE (n = 55) using Spearman rank correlation. Each point represents one patient. The blue line represents the linear fit. Spearman ρ = -0.57, p < 0.001.
